# Supplementary material for: Traffic light optimization with low penetration rate vehicle trajectory data
Source: Nat Commun. 2024 Feb 20;15:1306. doi: 10.1038/s41467-024-45427-4 (PMC10879128; doi:10.1038/s41467-024-45427-4)
Supplement: Supplementary file 1 — Supplementary Information [file 41467_2024_45427_MOESM1_ESM.pdf]

Supplementary Information

Traffic light optimization with low penetration rate vehicle trajectory data

Xingmin Wang[1], Zachary Jerome[1], Zihao Wang[1], Chenhao Zhang[2], Shengyin Shen[3], Vivek Vijaya Kumar[4], Fan Bai[4], Paul Krajewski[4], Danielle Deneau[5], Ahmad Jawad[5], Rachel Jones[5], Gary Piotrowicz[5], Henry X. Liu[1,3,6,*]

[1] *Department of Civil and Environmental Engineering, University of Michigan, Ann Arbor, MI, 48105, USA*
[2] *Department of Computer Science and Engineering, University of Michigan, Ann Arbor, MI, 48105, USA*
[3] *University of Michigan Transportation Research Institute, University of Michigan, Ann Arbor, MI, 48105, USA*
[4] *General Motors Research & Development, Warren, MI, 48092, USA*
[5] *Road Commission for Oakland County, Beverly Hills, MI, 48025, USA*
[6] *Mcity, University of Michigan, Ann Arbor, MI, 48105, USA*
[*] *Corresponding Author, henryliu@umich.edu*

Contents

1 Notations used in the paper **2**

2 Related works **2**
2.1 Stochastic traffic flow models . . . 2
2.2 Traffic state estimation with vehicle trajectory data . . . 4
2.3 Traffic signal control and optimization . . . 4

3 Probabilistic time-space (PTS) diagram with residual queue **6**
3.1 Discrete queueing model with residual queue . . . 6
3.2 PTS diagram with residual queue . . . 8

4 Additional details of the queueing model **9**
4.1 Effective green time . . . 9
4.2 Permissive movements . . . 10
4.3 Approximation of a network of movements . . . 13

5 Pre-determined and calibrated parameters **14**
5.1 Saturation flow rate estimation . . . 14
5.2 Other parameters . . . 16

6 **Calculation of the stationary queue length distribution** 17

7 **Traffic signal optimization algorithms** 18
7.1 Traffic signal timing parameters and optimization 18
7.2 Iterative gradient-based optimization for isolated intersections 19
7.3 Coordinate-descent offset optimization 20

8 **Performance evaluation of selected intersections** 22

9 **Field implementation at isolated intersections** 22

1 Notations used in the paper

Please refer to Supplementary Table 1 for the notations and associated meanings used in the paper.

2 Related works

2.1 Stochastic traffic flow models

Traffic flow models play an important role in traffic state estimation, prediction, and traffic control. Although data-driven methods have received much attention in recent years[1,2], model-based methods are more reliable and interpretable, especially with missing data or incomplete observations. Commonly available traffic data cannot provide a complete observation of the overall traffic state: detector-based data and vehicle trajectory data are limited by detection availability (installation) and penetration rate, respectively. In this case, it is important to incorporate traffic flow models as the prior knowledge for data assimilation[3]. Other than traffic state estimation, traffic flow models are also critical for model-based traffic control and optimization where the traffic state can be predicted under different traffic signal parameters[4,5].

Traffic flow models have different scales from microscopic to macroscopic. These scales have different applications with the trade-off being between model accuracy and computational efficiency. This paper focuses on a macroscopic traffic flow model for signalized intersections where each road segment or movement is the basic element. A movement is defined as a certain moving direction through the signalized intersection (e.g., through, left turn). First-order models like the Lighthill-Whitham-Richards (LWR) model[6,7] are the most commonly used traffic flow models for urban traffic networks and are significantly influenced by intersections. Many different versions and formulations have been proposed based on the LWR model, including the cell and link transmission models[8,9], variational formulation[10,11], and Hamilton-Jacobi based formulations[12].

In addition to LWR-based models, there are other traffic flow models that can be used to model urban traffic networks with signalized intersections. Compared with LWR models, which are usually referred to as physical or spatial queue models, point-queue models have simpler traffic state representations and dynamics because they ignore vehicle lengths. For example, Aboudolas et al.[5] proposed the store-and-forward model as well as different traffic signal optimization formulations based on it. Due to its simplicity, it is also used by most pressure-based control methods for the theoretical derivation of network stability[13–15]. Queueing models are another family of point-queue models which are more often used to study steady-state traffic performance[16–21]. Besides the point-queue model, Wu and Liu[22] proposed a shockwave profile model by tracking the different shockwaves of each movement.

Supplementary Table 1: Notations used in the paper.

Notation	Meaning	Unit
Δt	Time interval	sec
Δu	Unit traffic flow per time interval	veh
(t, n)	Newellian coordinates (free-flow arrival time, unit traffic flow)	$(\Delta t,\ \Delta u)$
(t', s')	Normal time-space coordinates	(sec, meter)
h	Jam space headway per unit traffic flow	meter/Δu
v_f	Free-flow speed	meter/sec
h_0	Jam space headway	meter/veh/lane
z	Number of lanes	1
q^{m}	Saturation flow rate	veh/lane/hour
(a^k, x^k, b^k)	Arrival time, stop location, and departure time for trajectory k	$(\Delta t, \Delta u, \Delta t)$
$X(t)$	Number of stopped vehicles at time t (point queue)	Δu
$X^{\mathrm{n}}(t)$	Location of the last stopped vehicles at time t (spatial queue)	Δu
$\Psi_{t,t^r}(\cdot)$	Mapping function between point and spatial queue at time t	N/A
t^r	The end of the most recent red light	Δt
$A(t)$	Arrival at time t	$\{0, 1\} \cdot \Delta u$
$B(t)$	Departure at time t	$\{0, 1\} \cdot \Delta u$
$a(t)$	Probability that there is an arrival at time t	$[0, 1]$
$b(t)$	Probability that there is a departure at time t	$[0, 1]$
$x(t, n)$	Probability that the point queue $X(t)$ is n at time t	$[0, 1]$
$S(t)$	Traffic signal state at time t	$\{0, 1\}$
$\rho^{\mathrm{n}}(t, n)$	Probability that a unit traffic flow travels from $(t, n+1)$ to (t, n)	$[0, 1]$
$\rho^{\mathrm{t}}(t, n)$	Probability that a unit traffic flow travels from (t, n) to $(t, n+1)$	$[0, 1]$
ϕ	Penetration rate	%
$a^{\mathrm{obs}}(t), b^{\mathrm{obs}}(t)$	Observed number of arrival/departure at time t	veh
$a^{\mathrm{sc}}(t), b^{\mathrm{sc}}(t)$	Scaled arrival/departure at time t	1
$\hat{a}(t), \hat{b}(t)$	Estimated arrival and departure probability	$[0, 1]$
N_c	Number of cycles that are used to aggregate vehicle trajectories	1
$\bar{d}^{\mathrm{obs}}$	Average control delay from observed vehicle trajectories	sec
n^{obs}	Number of observed vehicle trajectories	veh
$\hat{d}(\phi)$	Model-estimated control delay given penetration rate ϕ	sec
$s \in \mathcal{S}$	Signal timing parameters and the corresponding feasible set	N/A
$I(s)$	Performance index of the system given a signal timing plan	N/A
$D(s)$	Total delay of the system given a signal timing plan	sec
$L(s)$	Total number of stops of the system given a signal timing plan	sec
o_j	Offset of the intersection j	sec
Δo_j	Relative offset between the intersection j and $j+1$	sec

Most of these traffic flow models, besides the queuing models, are deterministic. However, in the real world, both traffic demand and driving behavior are stochastic. Compared with deterministic traffic flow models, stochastic traffic flow models are more realistic and can be easily used for stochastic traffic state estimation with incomplete or flawed observations. Therefore, researchers have spent much effort developing different stochastic traffic flow models. For example, Sumalee et al.[23] and Flötteröd and Osorio[19] proposed the stochastic version of the cell and link transmission model, respectively. Jabari and Liu[24] proposed a stochastic traffic flow model in which the

randomness originated from the drivers' gap choice. Jabari and Liu[25] also derived the Gaussian approximation of the model and utilized it for traffic state estimation using loop detector data. While most of these models were established based on Eulerian coordinates, Zheng et al.[26] proposed a stochastic traffic flow model based on Lagrangian coordinates.

Although different stochastic traffic flow models have been proposed, they can hardly be used for traffic state estimation based on vehicle trajectory data with a certain penetration rate. In this case, most of the uncertainty in the system comes from unknown traffic demand instead of stochastic driving behavior[24–26]. Besides, LWR models like the cell transmission model already have a high dimension by splitting the roadway into cells. It becomes much more complicated when it is extended to a stochastic setting. This limits its applicability for other uses. Queueing models mainly consider stochastic demand as the origin of the uncertainty. However, as point-queue models, queueing models ignore the length of the vehicle and cannot directly model the spatial propagation or distribution of the vehicles, especially when there is over-saturation or a residual queue for certain traffic cycles.

2.2 Traffic state estimation with vehicle trajectory data

Readers can refer to[27] and[28] for a more complete review of traffic state estimation with vehicle trajectory data. Existing methods can be roughly divided into shockwave-based methods and statistical estimation methods. In shockwave-based methods, the basic idea is to detect the shockwave in the time-space diagram and use shockwave theory[6,7,29] to estimate the traffic state[30–32]. One of the typical works is from Cheng et al.[30], which used a classification method to detect the featured points when the observed trajectories change their motions to construct the shockwave in the time-space diagram and used the shockwave to estimate the cycle-by-cycle queue length. Instead of directly constructing the shockwave by using the featured points from the observed trajectory, Ban et al.[31] and Hao et al.[32] used travel time to construct the shockwaves and estimate the queue lengths and the signal timing plan.

In stochastic estimation based on the stop locations of the connected vehicles, the observed stopped connected vehicles at certain snapshots are the input for different statistical estimation methods (maximum likelihood estimation or Bayesian estimation) which estimate the unknown parameters or states[33–39]. These methods are derived based on different assumptions such as the Poisson[35] or general arrival[38] processes and independent[38] or correlated[39] queue length distribution. The intuition of these studies is similar even though different assumptions are adopted. Compared with deterministic estimation methods[31,40,41], stochastic estimation methods can better utilize prior information and also provide stochastic estimation results including estimation uncertainty. However, most of the existing stochastic estimation methods only look into the stop location patterns at certain snapshots[33,37–39,42]. They did not perform estimation based on a stochastic traffic flow model and hence cannot be used for traffic state prediction.

2.3 Traffic signal control and optimization

There are many survey papers on traffic signal control and optimization[27,43,44]. Besides, readers can also refer to the traffic signal manual[45] for a more comprehensive introduction.

According to the responsiveness and complexity, traffic signal control systems can be divided into three categories: 1) fixed-time; 2) vehicle-actuated; and 3) adaptive control. Fixed-time control is usually used by intersections without any detection capability. For these intersections, traffic signal timing parameters are pre-determined and optimized by using offline historical data. Many offline tools, such as SYNCHRO[46], TRANSYT-7F[47], and PASSERTM V[48], can be used to generate

the offline signal timing parameters. However, the main limitation of fixed-time control is that it cannot respond to the time-varying traffic demand. Vehicle-actuated control overcomes this problem by applying a more responsive rule-based strategy using the data from detectors[45]. The vehicle-actuated control keeps the same phase if the headway between vehicles is less than a certain threshold, subjecting to the minimum and maximum green at the same time.

Compared with vehicle-actuated control which is a rule-based control with fixed parameters, adaptive traffic signal control is usually built based on certain traffic models and parameter selection (optimization) programs. Therefore, it is more flexible and complicated. The most commonly used adaptive signal control systems in the world include SCOOT[49] and SCATS[50]. Besides, there are also other adaptive traffic signal control systems such as OPAC and RHODES[51,52]. However, such adaptive signal control systems are not commonly deployed due to the computational complexity and hardware installation requirements[53].

During the past decades, traffic signal control and optimization have continued to draw attention from researchers. Different methods have been used for both fixed-time and real-time traffic signal optimization, including rule-based methods, model-based optimal control methods, and RL methods, etc. The vehicle-actuated control, as aforementioned, is a rule-based control method with given pre-determined parameters. Except for the rule-based methods, optimization-based methods are also frequently used to determine traffic signal parameters or states. Rolling-horizon optimization (i.e., receding-horizon optimization, model predictive control) has been widely used to formulate the real-time traffic signal control problem[4,54,55], which minimizes the total delay of the system while subjecting to the traffic flow model and traffic signal constraints.

Reinforcement Learning (RL) has become a popular approach for traffic signal control, as evidenced by several studies[43,56–60]. RL can directly learn an end-to-end control policy from the observation by interacting with the simulation environment iteratively. Most of the existing literature using RL for traffic signal control focuses on the design of the input state space and reward[43] while utilizing different RL techniques such as the multi-agent algorithms[59]. Despite the abundance of research utilizing RL for traffic signal optimization, there remains a significant gap between research and implementation in the real world. One of the main concerns is the reliability of RL-based approaches. RL controllers trained offline in a simulation environment may not perform well in real-world scenarios due to the limited fidelity of the simulation. On the other hand, training RL controllers directly in the real world raises additional challenges, particularly in managing the risks associated with exploration during the learning process.

The development of connected and automated vehicles (CAV) brings new challenges and opportunities for traffic signal control and optimization. Both automated vehicles (AV) and connected vehicles (CV) could serve as mobile sensors, which provide data such as vehicle trajectories and their observations (for AVs with detection ability) that can be used to optimize the traffic signal operation[61]. Moreover, AVs have the potential to serve as moving regulators to further improve the stability of the traffic flows[62–64]. Readers can refer to survey papers[27,44] for a more comprehensive review. Although many studies have proposed different signal control methods with CAV and demonstrated promising results in the simulation environment, they can hardly be used in the field since the current market penetration rates for both CV and AV are much less than what is assumed in these research studies.

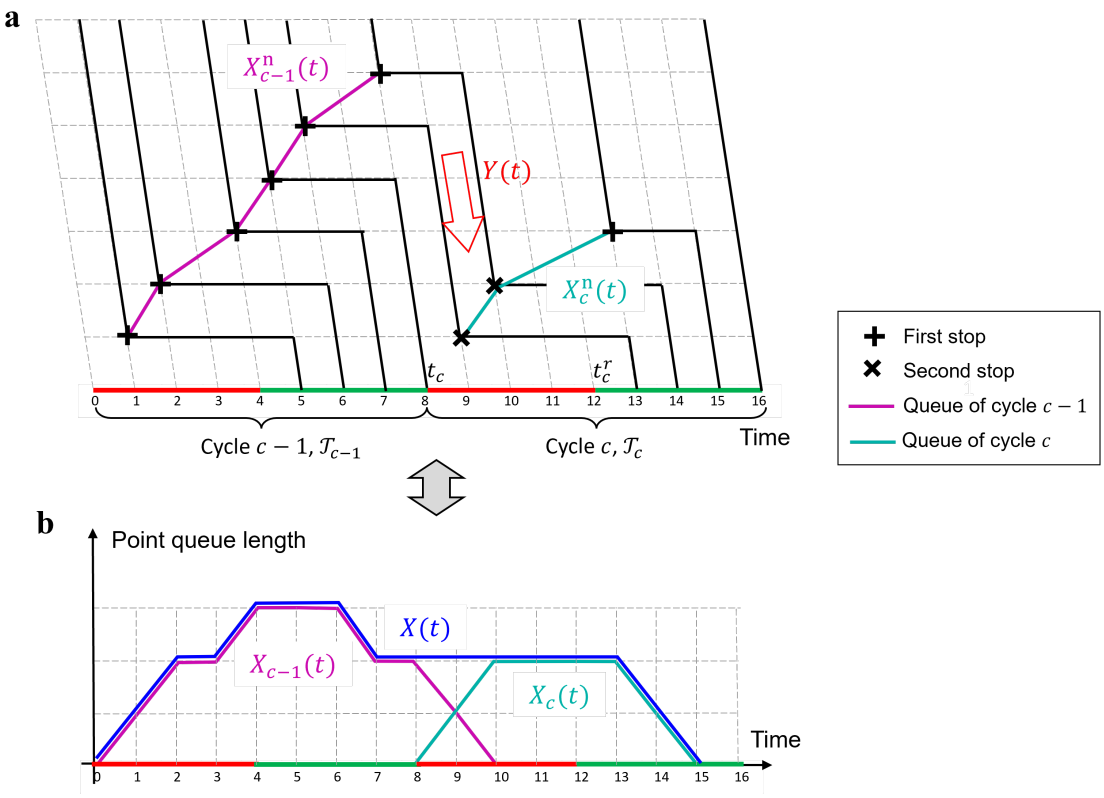

Supplementary Figure 1: Point-queue representation of vehicle trajectories with a residual queue at the end of the cycle. (a) $X^{\mathrm{n}}_c(t)$ denotes the spatial queue length of cycle c at time t. There will be a residual queue at the end of the cycle if the queue is not fully discharged during the green light. $Y(t)$ refers to the internal flow that leaves the queue of the previous cycle and joins the latest cycle. (b) Point queue length $X_c(t)$ and $X_{c-1}(t)$ that correspond to the vehicle trajectories. $X(t)$ denotes the total point queue length.

3 Probabilistic time-space (PTS) diagram with residual queue

3.1 Discrete queueing model with residual queue

Methods in the main paper demonstrated how the discrete queueing model can be mapped to the probabilistic time-space diagram without considering any residual queue or over-saturation. To make the model more generic so that it can deal with these cases, we need to keep track of the queue within each individual cycle. Supplementary Figure 1 illustrates how to decompose the overall queue into queues for each individual cycle. A cycle c starts with a red light at time t_c and the queue length starts to increase. Let $X_c(t)$ and $X^{\mathrm{n}}_c(t)$ denote the point and spatial queue lengths of cycle c, respectively. For each cycle, the spatial and point queue lengths have the following mapping relationship:

$$X^{\mathrm{n}}_c(t) = \Psi_{c,t}(X_c(t)), \quad \text{where } \Psi_{c,t}(n) = \begin{cases} n + (t - t_c^r)^+, & n > 0 \\ 0 & n = 0 \end{cases}. \tag{S1}$$

where $\Psi_{c,t}(\cdot)$ is the mapping function that projects the point queue $X_c(t)$ to the spatial queue $X^{\mathrm{n}}_c(t)$. The mapping function $\Psi_{c,t}(\cdot)$ is the same as Equation (8), but we use a slightly different notation: Here we use the subscript c to specify the index of the cycle and different cycles have

different red light end times t_c^r. Let $X(t)$ represent the overall point queue length at time t. We have:

$$X(t) = X_c(t) + X_{c-1}(t), \tag{S2}$$

By using $X(t)$ as the overall queue length, it is easy to verify that the discrete queueing model given by Equation (5-7) in the main paper still holds. However, we are no longer able to get the spatial queue information from the overall queue length. The overall queue $X(t)$ needs to be decomposed into the different cycles so that the spatial queue can be derived according to Equation (S1). As shown in Supplementary Figure 1a, the queue length $X_c^{\mathrm{n}}(t)$ is essentially the downstream of the residual queue $X_{c-1}^{\mathrm{n}}(t)$ from the previous cycle and the internal flow $Y(t)$ denotes vehicles that depart the residual queue and join the new queue.

Let $\mathcal{T}_c$ be the set of time steps of c-th cycle, then for each cycle c and $t \in \mathcal{T}_c$, the discrete queueing model can be written as:

$$X_{c-1}(t) = X_{c-1}(t-1) + A(t) - Y(t) = X'_{c-1}(t) - Y(t) \tag{S3}$$

$$X_c(t) = X_c(t-1) + Y(t) - B(t) = X'_c(t) - B(t) \tag{S4}$$

where $Y(t)$ and $B(t)$ are determined by:

$$\mathbb{P}(B(t) = 1) = b(t) = \mathbb{P}(X'_c(t) \geq 1) \cdot S(t); \tag{S5}$$

$$\mathbb{P}(Y(t) = 1) \equiv y(t) = \mathbb{P}(X'_{c-1}(t) \geq 1) \cdot 1. \tag{S6}$$

The internal flow given by Equation (S6) can be considered to be controlled by a constant green light since the vehicles in the residual queue $X_{c-1}(t)$ are not blocked and will join the new queue $X_c(t)$ continuously. Supplementary Figure 2 shows the probabilistic graphical model by decomposing the queue length into different cycles (residual queue and queue of the current cycle). The left-hand-side figure shows the time steps within the cycle c while the right-hand-side figure shows the transition between different cycles. Note that we need to assume that the queue length will only extend to the following cycle. See Assumption 1 for more details and discussions.

Assumption 1 *The queue length of a cycle does not extend to the cycle after the following cycle. This assumption holds when the traffic volume is slightly larger than capacity for some of the cycles, which is true in most real-world cases. One simple counterexample of this assumption is a highly congested movement where some vehicles need to wait for more than 2 cycles to pass the intersection. The same method proposed in this paper can be used but it will lead to a more complicated formulation, and hence we do not spend effort repeating the same procedure here.*

By adding Equation (S3) and Equation (S4), we have:

$$\underbrace{X_c(t) + X_{c-1}(t)}_{X(t)} = \underbrace{X_c(t-1) + X_{c-1}(t-1)}_{X(t-1)} + A(t) - B(t) \tag{S7}$$

This is the same as Equation (5) in the main paper, which means the overall queue length $X(t)$ has the same transition but here it is decomposed into different cycles.

Let $x(t, k_r, k)$ represent the joint distribution of the residual queue $X_{c-1} = k_r$ and queue of the current cycle $X_c = k$. The transition of Equation (S3-S6) can be written as:

$$x'(t, k_r+1, k) = x(t-1, k_r, k) \cdot a(t) + x(t-1, k_r+1, k) \cdot (1 - a(t)) \tag{S8a}$$

$$x''(t, k_r-1, k) = x'(t, k_r, k+1), \; k_r \geq 1 \tag{S8b}$$

$$x(t, k_r, k) = x''(t, k_r, k+1) \cdot S(t) + x''(t, k_r, k) \cdot (1 - S(t)), \; k \geq 1 \tag{S8c}$$

$$x(t, k_r, 0) = x''(t, k_r, 1) \cdot S(t) + x''(t, k_r, 0) \tag{S8d}$$

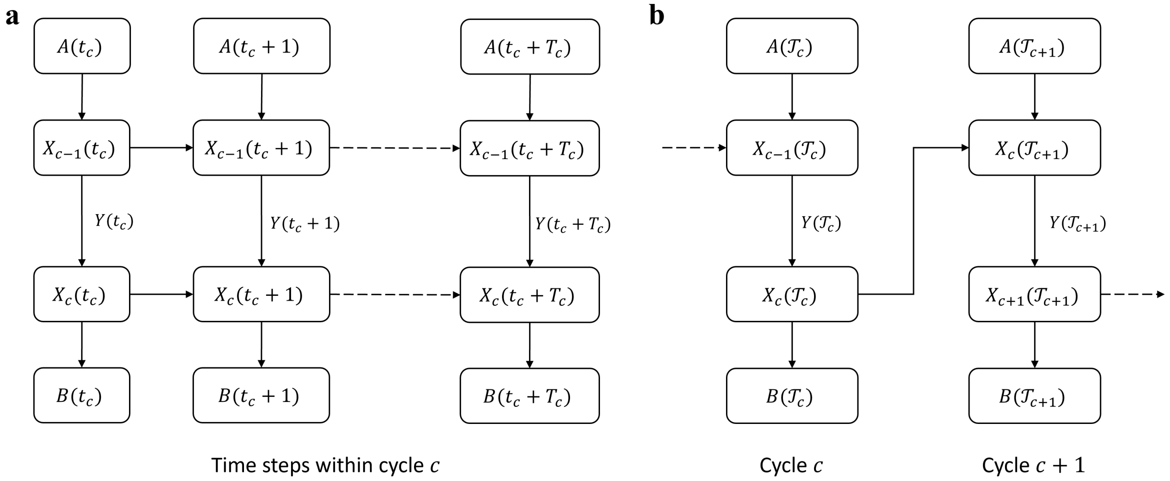

Supplementary Figure 2: Probabilistic graphical model with the residual queue. (a) Probabilistic graphical model within a cycle. For each time step, the new arrival will first join the residual queue from the previous cycle (if any), and then it goes to the queue of the current cycle through the internal flow $Y(t)$. (b) Probabilistic graphical model between cycles.

We also have the internal flow $y(t)$ and departure $b(t)$ determined by:

$$y(t) = \sum_{k_r=1}^{\infty}\sum_{k=0}^{\infty} x'(t, k_r, k) \tag{S9}$$

$$b(t) = \sum_{k_r=0}^{\infty}\sum_{k=1}^{\infty} x''(t, k_r, k) \cdot S(t) \tag{S10}$$

3.2 PTS diagram with residual queue

This subsection shows how to project the discrete queueing model to the corresponding PTS diagram when considering residual queues. As shown in Supplementary Figure 3, for each cycle c and time step $t \in \mathcal{T}_c = \{t_c, t_c+1, \ldots, t_c+T_c\}$, there are five different parts: 1) arrivals to the residual queue, 2) residual queue stop state, 3) internal flows from the residual queues to the new queues, 4) stop state of the queue of the current cycle, and 5) departures. The probability of each part is given below.

The arrival to the residual queue is similar to Equation (11) in the main paper. $\forall t \in \mathcal{T}_c$, we have:

$$\rho^{\mathrm{n}}\left(t, \Psi_{c-1,t}(n)\right) = \mathbb{P}(A(t)=1) \cdot \mathbb{P}\left(X_{c-1}(t) < n\right) = a(t) \cdot \sum_{k_r=0}^{n-1}\sum_{k=0}^{\infty} x(t, k_r, k) \tag{S11}$$

The residual queue stop state is similar to Equation (8) in the main paper. $\forall t \in \mathcal{T}_c$, we have:

$$\rho^{\mathrm{t}}\left(t, \Psi_{c-1,t}(n)\right) = \mathbb{P}(X_{c-1}(t) \geq n) = \sum_{k_r=n}^{\infty}\sum_{k=0}^{\infty} x(t, k_r, k). \tag{S12}$$

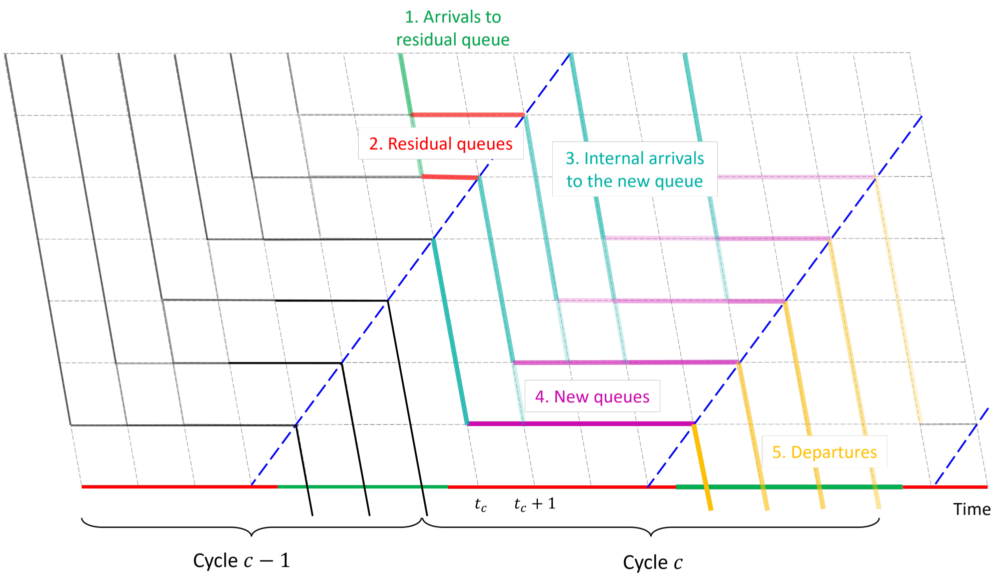

Supplementary Figure 3: Probabilistic time-space (PTS) diagram with a residual queue. When there is a residual queue at the end of the cycle, the line segments in the PTS diagram will have five different cases labeled by different colors.

The internal arrival to the new queue is given by ($\forall t \in \mathcal{T}_c$):

$$\rho^{\mathrm{n}}\left(t, \Psi_{c,t}(n)\right) = \mathbb{P}\left(X'_{c-1}(t) \geq 1 \ \&\ X_c(t) < n\right) = \sum_{k=1}^{\infty} \sum_{k=0}^{n-1} x'(t, k_r, k) \tag{S13}$$

Equation (S13) shows the probability that an internal flow departs from the residual queue and arrives at the new queue at location $X_c(t) = n$. It happens whenever the residual queue $X'_{c-1}(t)$ is not empty and the new queue $X_c(t)$ is less than n at the same time.

New queues:

$$\rho^{\mathrm{t}}\left(t, \Psi_{c,t}(n)\right) = \mathbb{P}(X_c(t) \geq n) = \sum_{k_r=0}^{\infty} \sum_{k=n}^{\infty} x(t, k_r, k) \tag{S14}$$

Final departures:

$$\rho^{\mathrm{n}}\left(t, 0 : \Psi_{c,t}(-1)\right) = \mathbb{P}(B(t) = 1) = b(t). \tag{S15}$$

4 Additional details of the queueing model

4.1 Effective green time

Due to the perception-reaction time (PRT) and vehicle acceleration after the green light starts, effective green time is known to be slightly different from the display green time. Similarly, after the green time ends, there is still a certain probability that some vehicles clear the intersection during the yellow time. Supplementary Figure 4 shows how the effective green time can be derived based on the raw signal phase and timing (SPaT) information. For this specific movement, the green time and yellow time are G and Y, respectively. Let μ_g be the average PRT while $\mu_y = G + Y/2$ is halfway through the yellow time. Here we will show two different methods to get effective green time under different uses.

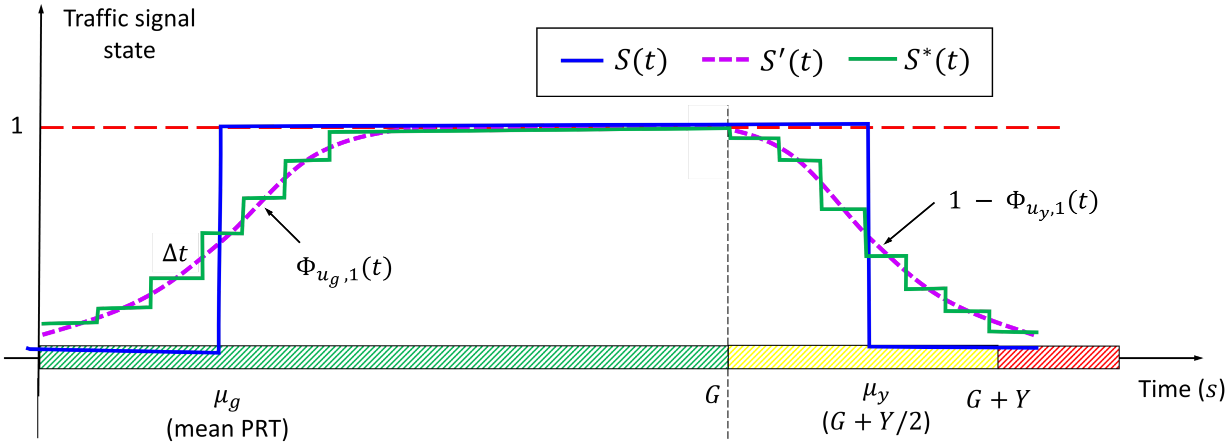

Supplementary Figure 4: Effective green time. The x-axis plots the signal light indication given by the traffic signal. The binary effective green time $S(t)$, used for the PTS diagram, is shown by the blue line and is parameterized by the average PRT μ_g and the halfway point of the yellow time μ_y. The effective green time $S'(t)$, calculated according to a cumulative Gaussian to model the green start-up time and vehicle behavior during the yellow light interval, is shown by the purple dashed curve. The discrete approximation $S^*(t)$ of $S'(t)$ is displayed with the green line.

The generation of the PTS diagram requires a deterministic binary traffic signal state. Therefore, a rectangular effective green time $S(t)$ will be used in this case which is determined by:

$$S(t) = \begin{cases} 1 & t \in [\mu_g, \mu_y] \\ 0 & \text{otherwise} \end{cases} . \tag{S16}$$

If we only care about the point-queue representation (arrival/departure profile, delay, etc.) of the movement without requiring the PTS diagram, the traffic signal state can also be a decimal number. We can use a cumulative Gaussian to model the green start-up time and the yellow light interval as shown by the purple curve $S'(t)$ as shown in Supplementary Figure 4. Let $\Phi_{\mu,\sigma^2}(x)$ be the cumulative density function (cdf) of a Gaussian distribution with mean μ and variance σ^2, $S'(t)$ is determined by:

$$S'(t) = \begin{cases} \Phi_{\mu_g,\sigma^2}(t) & t \le G \\ 1 - \Phi_{\mu_y,\sigma^2}(t) & t > G \end{cases} . \tag{S17}$$

The variance σ^2 is chosen as 1. Since the time is discrete with interval Δt, let $S^*(t)$ be the discrete approximation of $S'(t)$:

$$S^*(t) = \frac{1}{\Delta t} \int_{\tau=t}^{t+\Delta t} S'(t) d\tau \tag{S18}$$

Supplementary Figure 5 demonstrates some real world examples of $S(t)$ and $S^*(t)$ and how the predicted departures for both methods compare to the observations. The x-axis plots the signal light indication given by the traffic signal while the dashed green lines indicate the calculated signal states for each method. The model is able to match the observed departures fairly well with both methods, but $S^*(t)$ is a little more detailed, particularly during the green start-up time.

4.2 Permissive movements

This subsection will introduce how we approximate the effective green time or traffic states of permissive movements that must yield to other protected movements. Two examples are shown in

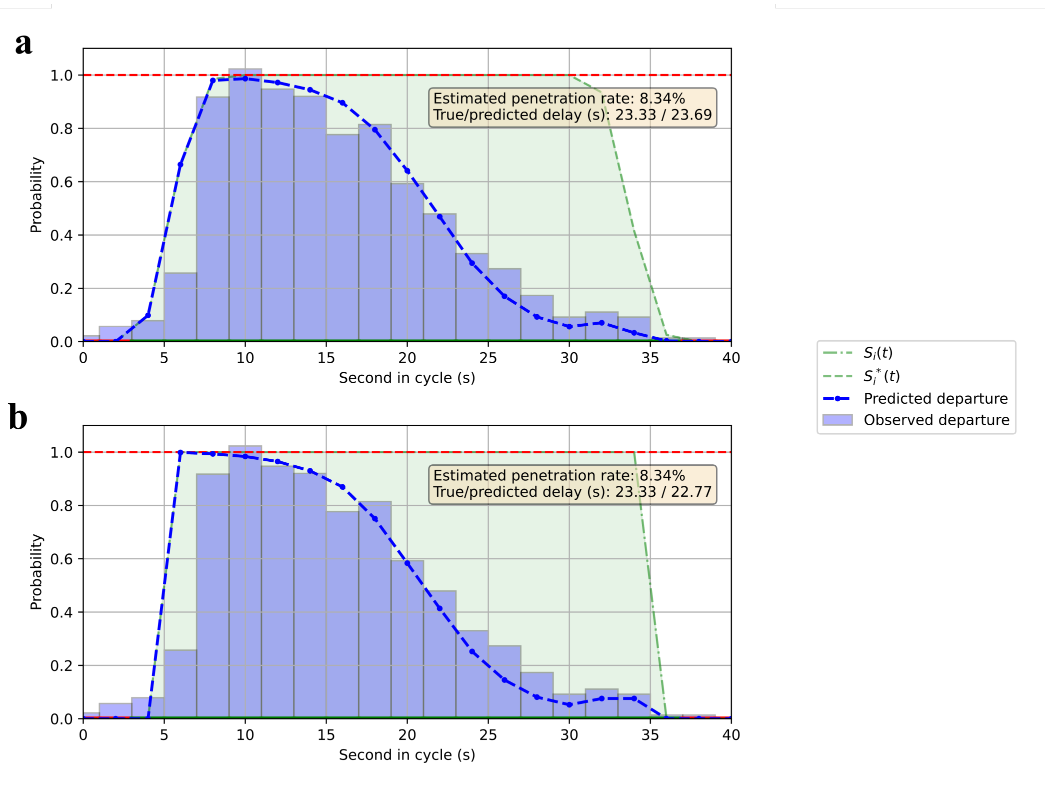

Supplementary Figure 5: Predicted vs. observed departures using $S^*(t)$ and $S(t)$. (a) Effective green time with discrete approximation of Gaussian distribution $S_i^*(t)$. The observed departure probability is shown by the shaded blue histogram. The predicted departure probability is illustrated by the dashed blue line. The dashed green line represents $S_i^*(t)$ and the shaded green area represents capacity. The estimated penetration rate, observed true delay, and predicted delay are reported in the tan box. (b) Binary effective green time $S_i(t)$. The dotted dashed green line represents $S_i(t)$. The estimated penetration rate, observed true delay, and predicted delay are reported in the tan box.

Supplementary Figures 6 and 7. In the first case (Figure 6), the left-turn and through movement from the opposing direction share the same green duration and the left-turn movement i needs to yield the opposing protected through movement p. In the second case (Figure 7), the right-turn movement i can turn right during the red time while yielding to the protected through movement p. We will only show the details of the first case. The intuition is to use a gap acceptance model to get the left-over capacity for the permissive movements after subtracting the through movement utilization.

As shown in Supplementary Figure 6, let $S_i^*p(t)$ and $B_p(t)$ represent the effective traffic signal states and the departure profile of the protected movement accordingly. The departure profile essentially represents the utilization of the traffic signal state of the movement. Define the $B_p^c(t)$ as the left-over capacity, we have:

$$B_p^c(t) = \begin{cases} S_p^*(t) - B_p(t) & t \leq G \\ 1 - B_p(t) & G < t \leq G + Y \end{cases} . \tag{S19}$$

We use $1 - B_p(t)$ to calculate the left-over capacity for the yellow time since left-turn vehicles can usually clear the intersection during the entire yellow time. Instead of directly using the left-over

capacity as the traffic state for the permissive movement, a gap acceptance model is further applied since vehicles in the permissive movement might require the protected movement to be empty for a few consecutive time steps. Let ζ be the number of time steps of the gap acceptance model. The effective traffic signal state of the permissive movement is eventually determined by:

$$S_i^*(t) = \prod_{\tau=t+1-\zeta}^{t} B_p^c(\tau). \tag{S20}$$

We can apply the gap acceptance model to get the traffic signal states for right turn on reds in Supplementary Figure 7.

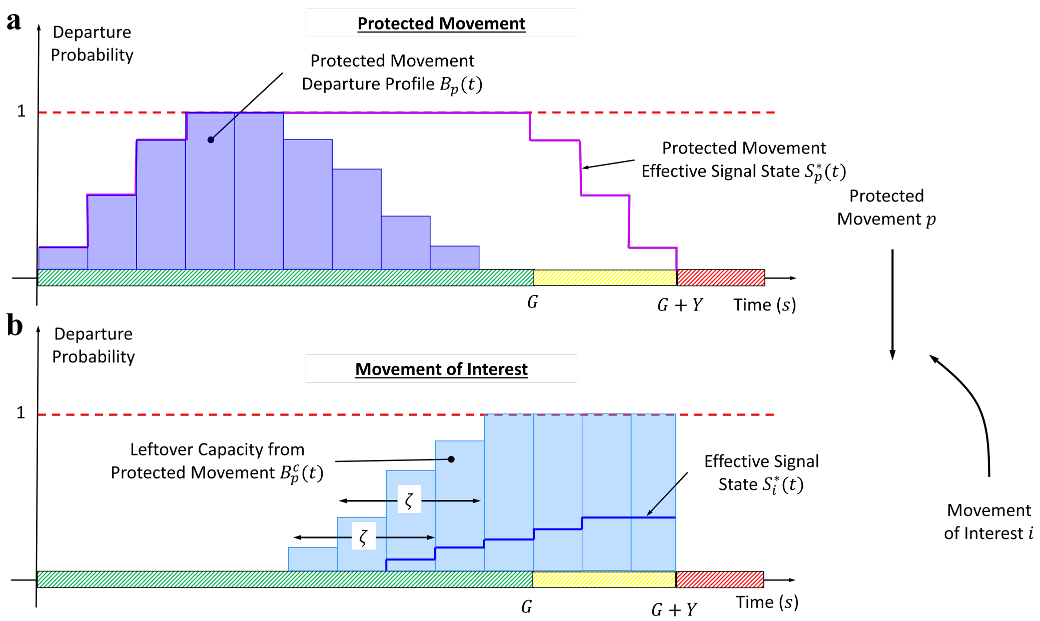

Supplementary Figure 6: Illustration of permissive left turn movement modeling. (a) Departure profile for the protected through movement p (blue shaded area). The effective signal state $S_p^*(t)$ is drawn in purple. (b) Effective signal state $S_i^*(t)$ for the left turning movement i (drawn in blue). The leftover capacity from the protected movement is plotted in light blue.

The real-world example in Supplementary Figure 8 illustrates how this method can accurately capture the observed departure profiles and the measured delays. The intersection analyzed in this figure is controlled by two phases, one for each street. As a result, the left-turn movement and the oncoming protected through movement share the same SPaT info and left turning vehicles must wait for a reasonable gap in the protected movement departures before proceeding through the intersection. When considering the protected movement, the predicted departure profile resembles the observed departure profile because it doesn't immediately allow vehicles to clear the intersection. One limitation of this model is that it will usually predict zero departures in the early stages of the green time because the model will predict maximum protected departures when the queue is first released (there is no leftover capacity when the light first turns green). Left turn departures could happen earlier in the green time during some random cycles where the conflicting movement's queue is small, but this is a rare occurrence and does not impact the model's ability to capture the average traffic state of the movement.

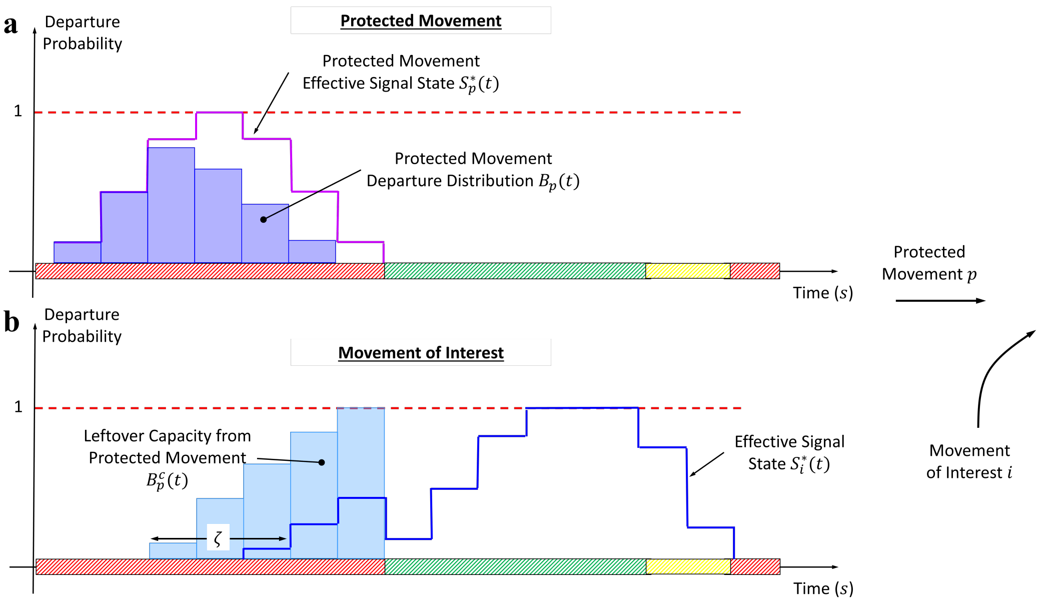

Supplementary Figure 7: Illustration of right turn on red modeling. (a) Departure profile for the protected through movement p (blue shaded area). The effective signal state $S_p^*(t)$ is drawn in purple. (b) Effective signal state $S_i^*(t)$ for the left turning movement i (drawn in blue). The leftover capacity from the protected movement is plotted in light blue.

4.3 Approximation of a network of movements

We also use single-queue decomposition approximation to model a general network consisting of multiple movements. For a movement within a traffic network as shown in Supplementary Figure 9, the arrival can be decomposed into the external arrival coming from external demand and the internal arrival from upstream movements. The arrival is determined by:

$$\mathbb{P}\left(A_i(t) = 1\right) = \sum_{k \in \mathcal{M}_i^u} \left(\mathbb{P}(B_k(t - T_{ki}) = 1) \cdot r_{ki} \frac{\Delta u_k}{\Delta u_i} \right) + \mathbb{P}(E_i(t) = 1) \tag{S21}$$

where $\mathcal{M}_i^u$ is the set of upstream movements of movement i, r_{ki} is the turning ratio, T_{ki} is the free-flow travel time from movement k to movement i; $E_i(t)$ is the exogenous arrival. Δu_i is the unit flow of movement i which is defined in the main paper as the saturation flow within the time interval Δt. The arrival given by Equation (S21) is also assumed to follow a Bernoulli distribution and arrivals at different time steps are also independent. Based on this assumption, the whole network is then decomposed into a set of movements; the stationary distribution of each movement queue length can be calculated according to the network topology from upstream to downstream.

Note that the arrival coming from the upstream is actually correlated and will be dependent on all the previous states[20,65]. Therefore, the actual stationary distribution of a network is hard to obtain, and the proposed method is a single-queue decomposition approximation. Besides, the upstream platoon might also disperse when vehicles travel along the link[66] and the downstream vehicles might also block the upstream vehicles[18]. In this paper, we use the simplest approximation without considering these complicated scenarios.

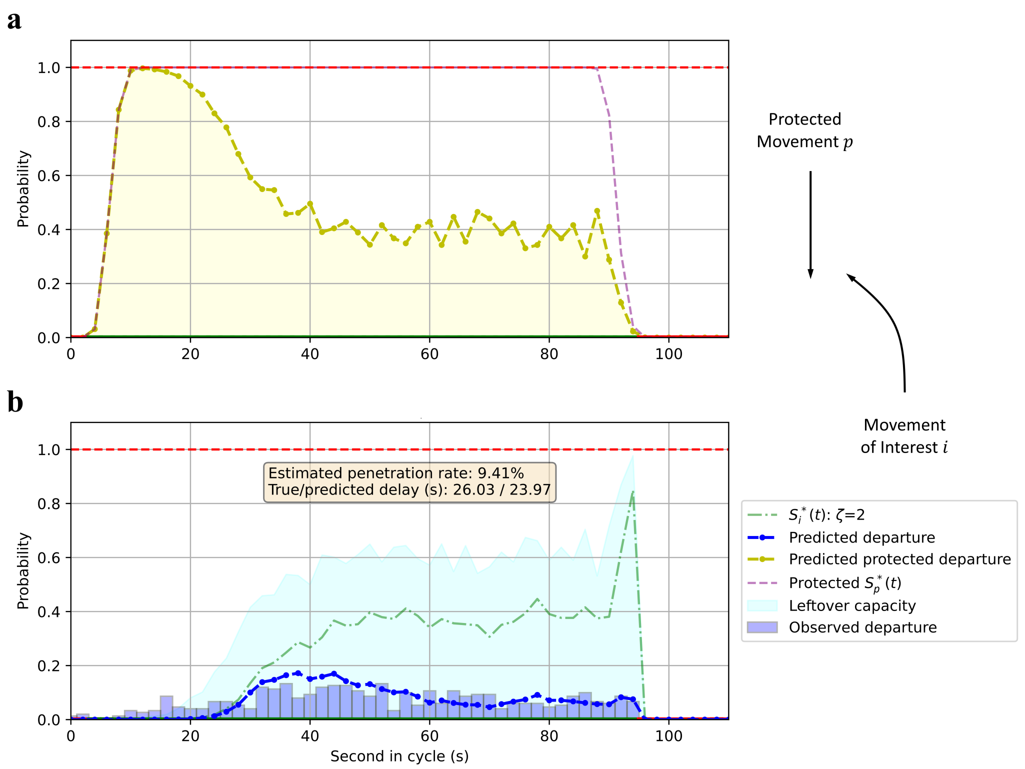

Supplementary Figure 8: Permissive left turn movement example: Quarton Road and Cranbrook RD WBL movement - PM TOD. (a) Protected movement predicted departure (dashed yellow line). The protected movement effective signal state $S_p^*(t)$ is drawn by the dashed purple line. (b) Leftover capacity (shaded light blue area) and resulting $S_i^*(t)$ (dotted dashed green line) for movement of interest. The observed departure is plotted in blue. The predicted departure is drawn with a dashed blue line. The estimated penetration rate, observed true delay, and predicted delay are reported in the tan box.

5 Pre-determined and calibrated parameters

5.1 Saturation flow rate estimation

The saturation flow rate q_i^{m} for each movement $i \in \mathcal{M}$ in the traffic network can be estimated from the vehicle trajectory data. For each trajectory k, the departure time after the green start t^k and queue distance q^k are illustrated by Supplementary Figure 10a. By assuming that the jam space headway is h_0, there will be q^k/h_0 vehicles in the queue, and this means that it takes time t^k to allow q^k/h_0 vehicles to clear the intersection. The saturation flow rate can then be estimated according to the following equation:

$$q_i^{\mathrm{m}} = \frac{\Delta n}{\Delta t} = \frac{\frac{\Delta q}{h_0}}{\Delta t} = \frac{\Delta q}{\Delta t} \cdot \frac{1}{h_0} \tag{S22}$$

where Δn is the number of vehicles clearing the intersection within the time interval Δt. The first equality is the definition of the saturation flow rate. $\Delta q/\Delta t$ is the slope of the $q - t$ scatter as

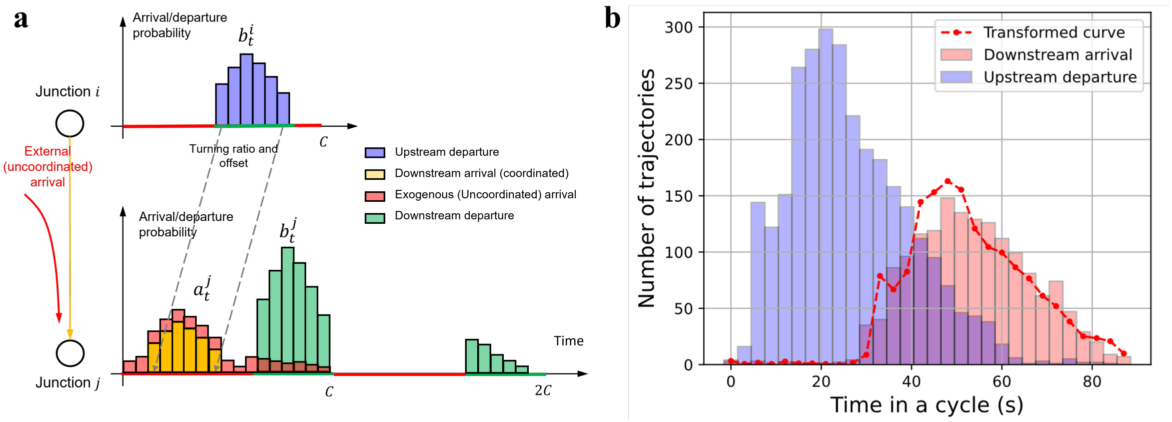

Supplementary Figure 9: Arrival of the coordinate movement. (a) Illustration of the arrival of a coordinated movement. The arrival might come from both the external arrival (uncoordinated) and coordinated arrival from the upstream intersection. (b) The downstream arrival (red bars) can be obtained by shifting and scaling the upstream vehicle departure (blue bars).

shown in Supplementary Figure 10b. This means that the saturation flow rate can be estimated through a linear regression over the $q - t$ scatters for all the collected trajectories. For a set of observations $\mathcal{O}_i = \{t_i^k, q_i^k, \forall k\}$ of movement i, we first eliminate trajectories that departed during the "start-up loss time". The RANSAC regression[67] is then used to further remove the outliers and get an accurate $q - t$ slope.

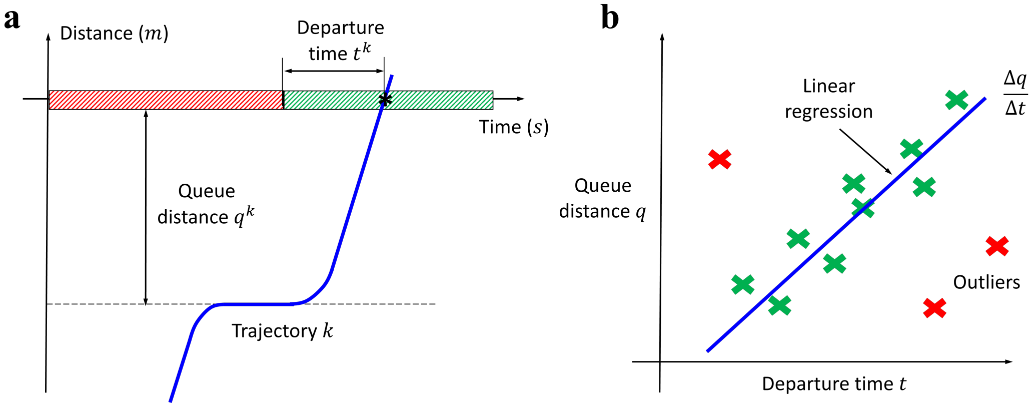

Supplementary Figure 10: Saturation flow rate estimation. (a) Queue distance and departure time. A hypothetical trajectory k with queue distance q^k and departure time t^k is drawn in blue. (b) $q - k$ scatters. The hypothetical linear regression line (slope of $\Delta q/\Delta t$) is plotted in blue. Inliers and outliers are plotted in green and red, respectively.

Supplementary Figure 11 illustrates an example of the saturation flow rate estimation where the estimated queue discharge rate is 3.54 m/s. Inlier points that are included in the final regression estimate are shown in green, while the outliers are shown in yellow. Around 92% of the total observed points were considered in the estimation. The R^2 value of 0.93 is a measure of linear regression accuracy. According to Equation (S22) and using an assumed jam space headway of 7 meters per vehicle, the estimated saturation flow rate of this movement will be 1,820 vphpl (vehicle

per hour per lane).

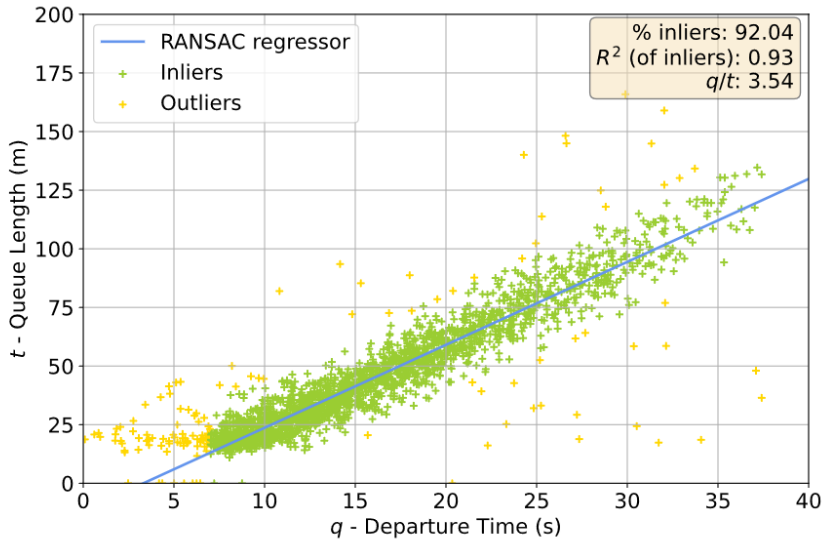

Supplementary Figure 11: Saturation flow rate estimation example: Maple Road and Adams Road WB movement - PM TOD. The RANSAC regression line is drawn in blue. The inlier and outlier scatters of the queue lengths and departure times are plotted in green and yellow, respectively. The percentage of inliers, R^2 of the regression line, and the slop $\Delta q/\Delta t$ are reported in the tan box.

5.2 Other parameters

Free-flow speed The free-flow speed of each movement is determined by the average free-flow speed extracted from each individual vehicle trajectory of the movement[68].

Turning ratios Since the trajectory data includes path information, the turning ratio can be estimated from the observed trajectories. For example, let movement j be the downstream of movement i, n_i^{obs} is the total number of observed trajectories of movement i while n_{ij}^{obs} is the total number of observed trajectories that travels to movement j from movement i, then the turning ratio r_{ij} is estimated as:

$$\hat{r}_{ij} = \frac{n_{ij}^{\text{obs}}}{n_i^{\text{obs}}}. \tag{S23}$$

Jam space headway Jam space headway refers to the space headway when vehicles stop at the signalized intersections. It is usually a constant determined by the vehicle length and the space between stopped vehicles (front bumper to front bumper). In practice, an average jam space headway of 7 meters per vehicle is usually used. In this paper, we manually adjust the jam space headway of each movement in the main paper Figure 4 slightly to improve the reconstructed PTS diagram.

Equivalent lane number One implicit assumption of the proposed model is that queues of different movements are separate and do not influence each other. However, this is not the case

when vehicles of different movements share the same lane. For example, it is very common that the right-turn movement and through movement share the same lane. In this case, we would still need to assume that queues of different movements are separate and do not interfere with each other. In this paper, we use the “equivalent lane number” as an approximation. For example, if there is a case that the through movement and the right-turn movement share a single lane and the traffic volume ratio is 3 : 1 (through : right-turn), then we will assign 0.75 lanes to the through movement and 0.25 lanes to the right-turn movements. However, this is just an approximation since different queues are still separate and the blocking between them cannot be modeled.

6 Calculation of the stationary queue length distribution

The following algorithm is consistent with the main paper which does not consider the residual queue mentioned in Supplementary Section 3. Equation (S25-S27) needs to be modified according to Equation (S8) if there is a residual queue at the end of the cycle.

Algorithm 1: Calculation of the stationary queue length distribution of a single movement

Input

- Arrival profile $\boldsymbol{a} = [a(1), a(2), \ldots, a(T)]$.
- Traffic signal state $\boldsymbol{s} = [s(1), s(2), \ldots, s(T)]$.
- Stopping criteria $\epsilon = 1e-6$.

Initiation:

- Queue length distribution $\boldsymbol{X}^0 \in \mathbb{R}^{T\times N}$ of iteration 0 where $x^0(t,k)$ is the probability that queue length at time step t is k. To start with, the queue is empty at $t=0$: $x^0(0,0)=1, x^o(0,k)=0, k\geq 1$;
- Departure profile $\boldsymbol{b}^0 = [b^0(1), \ldots, b^0(T)]$ at iteration 0 and $b(t)^0 = 0, \forall t \in \{1, \ldots, T\}$.

while *for iteration* $i = 0, 1, 2, ...$ **do**

Initiate the queue length distribution at the start of the cycle:

$$x^{i+1}(0,k) = x^i(T,k), \quad \forall n \tag{S24}$$

for *time in cycle* $t = 1, 2, ..., T$ **do**

Update the queue length distribution after new arrival:

$$x^{i+1}(t,k+1)' = x^{i+1}(t-1,k)\cdot a(t) + x^{i+1}(t-1,k+1)\cdot(1-a(t)), \quad \forall k \tag{S25}$$

Update the queue length distribution after new departure:

$$x^{i+1}(t,k) = x^{i+1}(t,k+1)'\cdot s(t) + x^{i+1}(t,k)'\cdot(1-s(t)), \quad \forall k\geq 1 \tag{S26a}$$

$$x^{i+1}(t,0) = x^{i+1}(t,1)'\cdot s(t) + x^{i+1}(t,0)' \tag{S26b}$$

Get the departure probability $b(t)$:

$$b^{i+1}(t) = \left(\sum_{k=1}^{N} x^{i+1}(t,k)'\right)\cdot s_i(t) \tag{S27}$$

if $\|\boldsymbol{X}^{i+1} - \boldsymbol{X}^i\|_F \geq \epsilon$ **then**

Set $\boldsymbol{b} = \boldsymbol{b}^{i+1}$, $\boldsymbol{X} = \boldsymbol{X}^{i+1}$ and terminate the iteration.

Return: departure probability $\boldsymbol{b}$ and queue length distribution $\boldsymbol{X}$.

7 Traffic signal optimization algorithms

7.1 Traffic signal timing parameters and optimization

Supplementary Figure 12 shows the main parameters of the fixed-time traffic signals, including the time of day (TOD) plans, cycle, splits, and offsets of each TOD. Let $\boldsymbol{s}$ represent the traffic signal parameters:

$$\boldsymbol{s} = \left\{\boldsymbol{\tau} = [\tau^1, \tau^2, \ldots, \tau^{K-1}], \{s^k = (C^k, \boldsymbol{g}^k, \boldsymbol{o}^k)\}\right\}, \tag{S28}$$

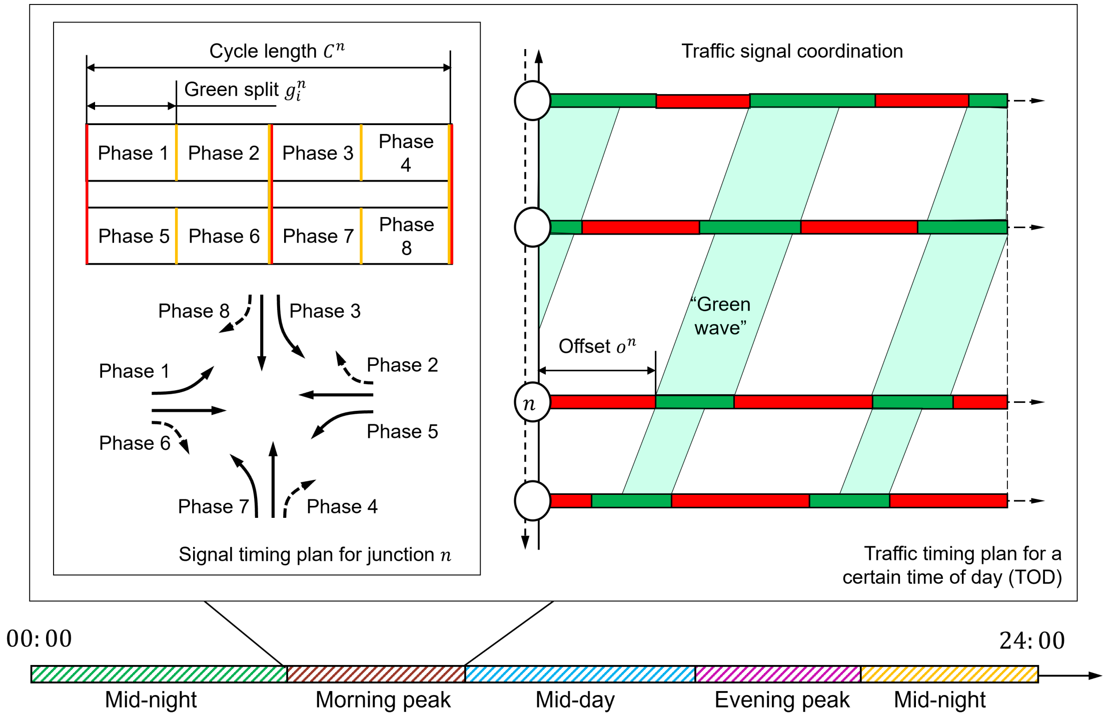

Supplementary Figure 12: Traffic signal timing parameters (fixed-time). The signal timing plan is split into different times of day (TODs). Within a certain time of day (TOD), the main parameters for intersections include the cycle length and green split. The offset determines the coordination of multiple intersections in a corridor.

where $\boldsymbol{\tau}$ is the TOD splits and τ^k is the boundary between TOD k and $k+1$, K is the number of TODs; s^k refers to the signal timing plan of TOD k, including the common cycle C^k, green splits for each movement $\boldsymbol{g}^k$, and offset for each intersection $\boldsymbol{o}^k$. Then the all-day performance index is composed of all TOD intervals:

$$I(\boldsymbol{s}) = \sum_{k=1}^{K} I^k(\boldsymbol{s}) = \sum_{k=1}^{K} \left[D^k(s^k) + \alpha L^k(s^k) \right] \tag{S29}$$

where the performance index of each TOD k is composed of the total estimated delay $D^k(\cdot)$ and the total estimated number of stops $L^k(\cdot)$. Given the calibrated traffic demand, both the total estimated delay and stops are determined by the traffic signal parameters S^k. The traffic signal optimization problem can be formulated as:

$$\boldsymbol{s}^* = \underset{\boldsymbol{s}}{\operatorname{argmin}}\, I(\boldsymbol{s}) \tag{S30}$$

which finds the optimal traffic signal parameters that minimize the overall delay and stops.

7.2 Iterative gradient-based optimization for isolated intersections

The gradient of the overall performance to the traffic signal parameters can be written as:

$$\nabla I(\boldsymbol{s}) = \sum_{k=1}^{K-1} \frac{\partial I(\boldsymbol{s})}{\partial \tau^k} d\tau^k + \sum_{k=1}^{K} \left[\frac{\partial I^k(S^k)}{\partial \boldsymbol{g}^k} d\boldsymbol{g}^k + \frac{\partial I^k(S^k)}{\partial C^k} dC^k + \frac{\partial I^k(S^k)}{\partial \boldsymbol{o}^k} d\boldsymbol{o}^k \right] \tag{S31}$$

Without having the closed analytical form, the partial derivative is estimated by numerical method. Taking the cycle length C^k of the kth TOD as an example, the partial derivative is given by:

$$\frac{I^k(\cdot)}{\partial C^k} = \frac{\hat{I}^k(\cdot, C + \Delta C) - I^k(\cdot, C)}{\Delta C} \tag{S32}$$

Equation (S32) has a clear physical meaning which quantifies how the system performance index changes by adding unit cycle length. Such gradient information can be used as an indication to the traffic signal diagnosis. The sign of the gradient indicates the direction that could improve the system performance while the magnitude of the gradient quantifies the potential benefits. As shown in Equation (S31-S32), the total derivative of the system performance can be decomposed into different terms with regard to different traffic signal parameters where their gradients are estimated separately. These gradients with clear physical meanings lead to different well-tagged diagnostic results including the green time imbalances, suboptimal cycle lengths, and inaccurate TOD splits.

Based on the diagnostic results given by these gradients, the traffic signal optimization is essentially a gradient-descent algorithm in the long run. For each iteration, roughly 2-3 weeks, new data is collected, and new gradients are estimated from the calibrated queueing model. The new signal timing plan will be based on the original timing plan and moves along the derivative direction for a certain step size. Note that it is not necessary to update all the traffic signal parameters each time, only those with large gradients. This is a simple yet practical and effective algorithm, especially for the update of the green split, cycle length, and TOD splits. They do not require major changes in most cases. The traffic patterns might also change over time and hence it is probably better to make minor adjustments each iteration while keeping the overall update process in the long term.

7.3 Coordinate-descent offset optimization

Offsets of intersections do not have much influence on the intersection capacity but could lead to better coordination among intersections. Therefore, unlike other traffic parameters which usually do not need to change much, major changes can be applied to the offsets. If we look into a specific TOD, the offset optimization problem can be formulated as:

$$\Delta \boldsymbol{o}^* = \underset{\Delta \boldsymbol{o}=[\Delta o_1,\ldots,\Delta o_{N-1}]}{\text{argmin}} \quad I(\Delta o_1, \Delta o_2, \ldots, \Delta o_{N-1}) \tag{S33}$$

where $I(\cdot)$ is the performance index (PI) of the calibrated traffic flow model which is determined by the relative offset vector $\boldsymbol{\Delta o} = [\Delta o_1, \ldots, \Delta o_{N-1}]$. Δo_j is the relative offset between intersection j and $j+1$ as shown in the main paper Figure 4e. Given the relative offsets $\boldsymbol{\Delta o}$, the offset o_j of intersection j can be determined as:

$$o_j = \left(\sum_{i=1}^{j-1} \Delta o_i \right) \mod T \tag{S34}$$

where T is the cycle length. The optimization problem given by Equation (S33) can be solved by a coordinate-descent algorithm. For each iteration i, relative offsets are optimized sequentially according to:

$$\Delta o_j^i = \underset{\Delta o_j}{\text{argmin}}\, I(\Delta o_1^i, \ldots, \Delta o_{j-1}^i, \Delta o_j, \Delta o_{j+1}^{i-1}, \ldots, \Delta o_{N-1}^{i-1}), \quad \forall j = \{1, 2, \ldots, N-1\} \tag{S35}$$

which can be solved through a line search program. This iterative program will stop when the improvement in the last iteration is less than a certain threshold.

Supplementary Table 2: Performance evaluation of selected signalized intersections.

Intersection	Avg. Control Delay (sec)	Avg. Number of Stops	Split-Failure Ratio (%)	Level of Service
AM (Morning Peak from 07:00 to 10:00)				
Adams Rd./Derby Rd.	16.46	0.490	2.80%	B
Adams Rd./Buckingham Rd.	10.86	0.405	1.20%	B
Adams Rd./E Maple Rd.	24.39	0.643	4.64%	C
Adams Rd. Bowers Rd.	10.28	0.340	1.48%	B
Adams Rd./E Lincoln St.	11.31	0.266	1.15%	B
Old Woodward Ave./Oakland Ave.	18.94	0.520	0.86%	B
Old Woodward Ave./Hamilton Rd.	9.56	0.182	0.88%	A
Old Woodward Ave./W Maple Rd.	14.36	0.314	1.08%	B
Old Woodward Ave./E Merrill St.	12.93	0.391	2.03%	B
Old Woodward Ave./E Brown St.	19.43	0.525	1.48%	B
Quarton Rd./Cranbrook Rd.	12.65	0.373	0.69%	B
E Lincoln St./Pierce St.	12.23	0.413	1.01%	B
MD (Mid-Day Period from 10:00 to 15:00)				
Adams Rd./Derby Rd.	16.42	0.465	2.65%	B
Adams Rd./Buckingham Rd.	9.23	0.305	1.31%	A
Adams Rd./E Maple Rd.	34.04	0.857	7.92%	C
Adams Rd. Bowers Rd.	10.56	0.313	2.18%	B
Adams Rd./E Lincoln St.	23.14	0.561	5.71%	C
Old Woodward Ave./Oakland Ave.	22.63	0.554	2.52%	C
Old Woodward Ave./Hamilton Rd.	10.11	0.240	1.04%	B
Old Woodward Ave./W Maple Rd.	21.06	0.475	4.70%	C
Old Woodward Ave./E Merrill St.	21.76	0.594	4.27%	C
Old Woodward Ave./E Brown St.	20.87	0.575	2.38%	C
Quarton Rd./Cranbrook Rd.	11.04	0.393	0.41%	B
E Lincoln St./Pierce St.	11.77	0.426	1.04%	B
PM (Evening Peak from 15:00 to 18:00)				
Adams Rd./Derby Rd.	10.03	0.387	0.42%	B
Adams Rd./Buckingham Rd.	8.43	0.356	0.33%	A
Adams Rd./E Maple Rd.	23.17	0.626	2.85%	C
Adams Rd. Bowers Rd.	12.43	0.385	2.46%	B
Adams Rd./E Lincoln St.	13.26	0.310	1.72%	B
Old Woodward Ave./Oakland Ave.	22.82	0.576	3.45%	C
Old Woodward Ave./Hamilton Rd.	13.15	0.343	2.73%	B
Old Woodward Ave./W Maple Rd.	21.83	0.499	4.67%	C
Old Woodward Ave./E Merrill St.	18.83	0.555	3.96%	B
Old Woodward Ave./E Brown St.	21.78	0.599	3.33%	C
Quarton Rd./Cranbrook Rd.	13.00	0.389	0.91%	B
E Lincoln St./Pierce St.	13.15	0.460	1.24%	B

8 Performance evaluation of selected intersections

Supplementary Table 2 shows the performance evaluation of those intersections that were implemented with new signal timing plans. It is generated based on three consecutive weeks' (only weekdays from Monday to Friday) data from 03/07/2022 to 03/25/2022 before the field implementation. For each intersection at each specific TOD period, the reported traffic performance metrics include the average control delay, average number of stops, split-failure ratio, and level of service (LOS). Average control delay and average number of stops are also used as the evaluation metrics by the before-and-after study in the main paper.

A vehicle trajectory is regarded as a split failure if it stops more than once before passing the intersection and the control delay is larger than a cycle. The split-failure ratio refers to the proportion of the split-failure trajectories to the total number of trajectories. A split-failure trajectory indicates that the green time is insufficient for a cycle and there is a residual queue at the end of the green time. Therefore, the split-failure ratio directly quantifies the risk of an intersection being over-saturated for some cycles. As shown in the table, Adams Rd. & E Maple Rd. has the largest split-failure ratio of up to around 8% during the mid-day period.

Traffic performance evaluation directly shows the traffic performance metrics that are calculated based on observed vehicle trajectory data. It can provide useful information such as where the congestion comes from, which intersection is the most congested intersection, and whether the intersection has a high risk of being over-saturated, etc. However, performance evaluation itself does not directly lead to true optimality gaps that can be improved by the re-timing of traffic signals. Compared with previous works that mainly focus on performance evaluation[68,69], this paper investigates more in-depth traffic modeling, state estimation, and traffic signal diagnosis/optimization.

Supplementary Table 2 is an intersection-level performance evaluation. The movement-level performance evaluation is also available.

9 Field implementation at isolated intersections

New signal timing plans were implemented at two intersections. The TOD boundary changes and their respective cycle lengths are shown in Supplementary Figure 13. All the parameter changes are reported in Supplementary Table 3 and Supplementary Table 4. The results for selected analysis periods are reported in Supplementary Table 5. At Quarton Rd. & Cranbrook Rd, a 4-second increase in the major split resulted in a 9.13% decrease in the PI during the AM TOD. The intersection also benefited from changing the PM TOD start time from 3:00 PM to 2:00 PM (results shown by the PM* analysis period). The increased cycle length during this hour resulted in a 21.45% reduction in the number of stops. The EVE TOD also experienced reductions in delay and the number of stops.

Lincoln Rd. & Pierce St. also experienced improvements in the PI for certain analysis periods. The boundary start change for the PM TOD resulted in a 9.83% reduction in the PI. However, a close look at Table 1a shows that the only parameters that changed during this time period were the green splits. The large improvement for this specific hour may indicate that new TODs may need to be formed as this hour improved much more compared to the MD and PM TODs that experienced the same change.

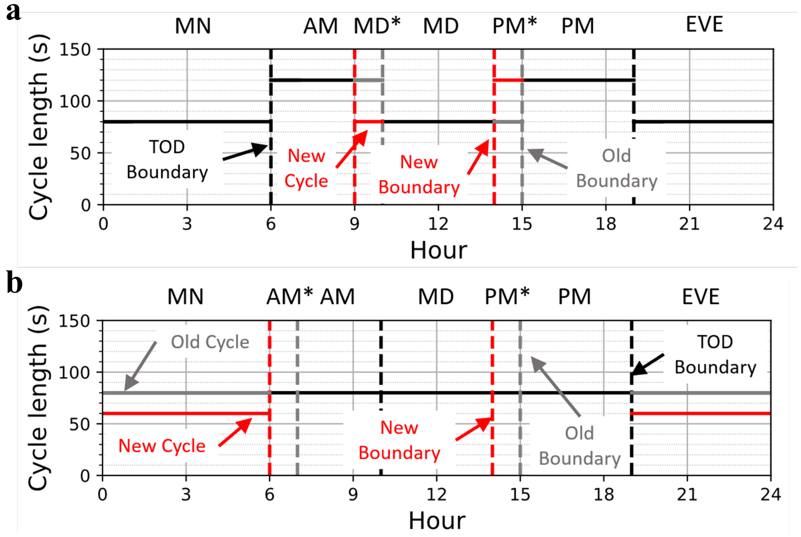

Supplementary Figure 13: TOD changes for isolated intersections. (a) Quarton Road and Cranbrook Road. Boundaries are drawn with dashed lines and cycle lengths are drawn with solid lines. Boundaries and cycle lengths that were kept, discarded, and added are drawn in black, grey, and red, respectively. (b) Lincoln Road and Pierce Street.

Supplementary Table 3: Quarton Rd. & Cranbrook Rd. Parameter Changes

TOD	Original Cycle	New Cycle	Original Major Split	New Major Split	Original Minor Cycle	New Minor Split
MN	80	80	54	56	26	24
AM	120	120	90	94	30	26
MD	80	80	54	56	26	24
PM	120	120	90	94	30	26
EVE	80	80	54	56	26	24

Supplementary Table 4: Lincoln Rd. & Pierce St. Parameter Changes

TOD	Original Cycle	New Cycle	Original Major Split	New Major Split	Original Minor Cycle	New Minor Split
MN	80	60	55	38	25	22
AM	80	80	55	55	25	25
MD	80	80	55	58	25	22
PM	80	80	55	58	25	22
EVE	80	60	55	38	25	22

Supplementary Table 5: Intersections performance table

Intersection	Analysis Period	Avg Delay (Before)	Avg Delay (After)	Delay % Change	Avg Stops (Before)	Avg Stops (After)	Stops % Change	PI % Change
Quarton Rd. & Cranbrook Rd.	06:00-09:00 (AM)	13.19	12.13	-8.03%	0.39	0.35	-9.51%	-9.13%
	14:00-15:00 (PM*)	12.65	12.24	-3.17%	0.45	0.36	-21.45%	-8.69%
	19:00-24:00 (EVE)	8.80	8.51	-3.29%	0.32	0.31	-3.09%	-3.34%
Lincoln Rd. & Pierce St.	10:00-14:00 (MD)	11.56	11.54	-0.18%	0.41	0.40	-4.46%	-1.32%
	14:00-15:00 (PM*)	12.43	11.48	-7.65%	0.46	0.41	-12.44%	-9.83%
	15:00-19:00 (PM)	12.71	12.57	-1.15%	0.45	0.45	-0.33%	-0.77%

References

1. Avila, A. M. & Mezić, I. Data-driven analysis and forecasting of highway traffic dynamics. *Nature communications* **11,** 2090 (2020).
2. Cui, Z., Ke, R., Pu, Z. & Wang, Y. Stacked bidirectional and unidirectional LSTM recurrent neural network for forecasting network-wide traffic state with missing values. *Transportation Research Part C: Emerging Technologies* **118,** 102674 (2020).
3. Wang, Y. *et al.* Real-time joint traffic state and model parameter estimation on freeways with fixed sensors and connected vehicles: State-of-the-art overview, methods, and case studies. *Transportation Research Part C: Emerging Technologies* **134,** 103444 (2022).
4. Lo, H. K. A novel traffic signal control formulation. *Transportation Research Part A: Policy and Practice* **33,** 433–448 (1999).
5. Aboudolas, K., Papageorgiou, M. & Kosmatopoulos, E. Store-and-forward based methods for the signal control problem in large-scale congested urban road networks. *Transportation Research Part C: Emerging Technologies* **17,** 163–174 (2009).
6. Light, M. & Whitham, B. On kinematic waves. I: Flow movement in long rivers; II: A theory of traffic flow on long crowed roads [C]. *Proceedings of Royal Society A,* 281–345 (1955).
7. Richards, P. I. Shock waves on the highway. *Operations research* **4,** 42–51 (1956).
8. Daganzo, C. F. The cell transmission model: A dynamic representation of highway traffic consistent with the hydrodynamic theory. *Transportation Research Part B: Methodological* **28,** 269–287 (1994).
9. Yperman, I., Logghe, S. & Immers, B. *The link transmission model: An efficient implementation of the kinematic wave theory in traffic networks* in *Proceedings of the 10th EWGT Meeting* **24** (2005).
10. Daganzo, C. F. A variational formulation of kinematic waves: basic theory and complex boundary conditions. *Transportation Research Part B: Methodological* **39,** 187–196 (2005).
11. Daganzo, C. F. A variational formulation of kinematic waves: Solution methods. *Transportation Research Part B: Methodological* **39,** 934–950 (2005).
12. Laval, J. A. & Leclercq, L. The Hamilton–Jacobi partial differential equation and the three representations of traffic flow. *Transportation Research Part B: Methodological* **52,** 17–30 (2013).
13. Varaiya, P. Max pressure control of a network of signalized intersections. *Transportation Research Part C: Emerging Technologies* **36,** 177–195 (2013).
14. Wang, X., Yin, Y., Feng, Y. & Liu, H. X. Learning the max pressure control for urban traffic networks considering the phase switching loss. *Transportation Research Part C: Emerging Technologies* **140,** 103670 (2022).
15. Levin, M. W. Max-Pressure Traffic Signal Timing: A Summary of Methodological and Experimental Results. *Journal of Transportation Engineering, Part A: Systems* **149,** 03123001 (2023).
16. Van Woensel, T. & Vandaele, N. Modeling traffic flows with queueing models: a review. *Asia-Pacific Journal of Operational Research* **24,** 435–461 (2007).
17. Viti, F. & Van Zuylen, H. J. Probabilistic models for queues at fixed control signals. *Transportation Research Part B: Methodological* **44,** 120–135 (2010).

18. Osorio, C. & Bierlaire, M. An analytic finite capacity queueing network model capturing the propagation of congestion and blocking. *European Journal of Operational Research* **196,** 996–1007 (2009).

19. Flötteröd, G. & Osorio, C. Stochastic network link transmission model. *Transportation Research Part B: Methodological* **102,** 180–209 (2017).

20. Boon, M. A. & van Leeuwaarden, J. S. Networks of fixed-cycle intersections. *Transportation Research Part B: Methodological* **117,** 254–271 (2018).

21. Oblakova, A. I. Queueing models for urban traffic networks (2019).

22. Wu, X. & Liu, H. X. A shockwave profile model for traffic flow on congested urban arterials. *Transportation Research Part B: Methodological* **45,** 1768–1786 (2011).

23. Sumalee, A., Zhong, R., Pan, T. & Szeto, W. Stochastic cell transmission model (SCTM): A stochastic dynamic traffic model for traffic state surveillance and assignment. *Transportation Research Part B: Methodological* **45,** 507–533 (2011).

24. Jabari, S. E. & Liu, H. X. A stochastic model of traffic flow: Theoretical foundations. *Transportation Research Part B: Methodological* **46,** 156–174 (2012).

25. Jabari, S. E. & Liu, H. X. A stochastic model of traffic flow: Gaussian approximation and estimation. *Transportation Research Part B: Methodological* **47,** 15–41 (2013).

26. Zheng, F., Jabari, S. E., Liu, H. X. & Lin, D. Traffic state estimation using stochastic Lagrangian dynamics. *Transportation Research Part B: Methodological* **115,** 143–165 (2018).

27. Guo, Q., Li, L. & Ban, X. J. Urban traffic signal control with connected and automated vehicles: A survey. *Transportation research part C: emerging technologies* **101,** 313–334 (2019).

28. Maripini, H., Khadhir, A. & Vanajakshi, L. Traffic State Estimation near Signalized Intersections. *Journal of Transportation Engineering, Part A: Systems* **149,** 03123002 (2023).

29. Newell, G. F. A simplified car-following theory: a lower order model. *Transportation Research Part B: Methodological* **36,** 195–205 (2002).

30. Cheng, Y., Qin, X., Jin, J., Ran, B. & Anderson, J. Cycle-by-cycle queue length estimation for signalized intersections using sampled trajectory data. *Transportation Research Record* **2257,** 87–94 (2011).

31. Ban, X. J., Hao, P. & Sun, Z. Real time queue length estimation for signalized intersections using travel times from mobile sensors. *Transportation Research Part C: Emerging Technologies* **19,** 1133–1156 (2011).

32. Hao, P., Ban, X., Bennett, K. P., Ji, Q. & Sun, Z. Signal timing estimation using sample intersection travel times. *IEEE Transactions on Intelligent Transportation Systems* **13,** 792–804 (2012).

33. Comert, G. & Cetin, M. Queue length estimation from probe vehicle location and the impacts of sample size. *European Journal of Operational Research* **197,** 196–202 (2009).

34. Comert, G. Queue length estimation from probe vehicles at isolated intersections: Estimators for primary parameters. *European Journal of Operational Research* **252,** 502–521 (2016).

35. Zheng, J. & Liu, H. X. Estimating traffic volumes for signalized intersections using connected vehicle data. *Transportation Research Part C: Emerging Technologies* **79,** 347–362 (2017).

36. Wong, W., Shen, S., Zhao, Y. & Liu, H. X. On the estimation of connected vehicle penetration rate based on single-source connected vehicle data. *Transportation Research Part B: Methodological* **126,** 169–191 (2019).

37. Zhao, Y. *et al.* Estimation of queue lengths, probe vehicle penetration rates, and traffic volumes at signalized intersections using probe vehicle trajectories. *Transportation Research Record* **2673,** 660–670 (2019).

38. Zhao, Y. *et al.* Various methods for queue length and traffic volume estimation using probe vehicle trajectories. *Transportation Research Part C: Emerging Technologies* **107,** 70–91 (2019).

39. Zhao, Y., Shen, S. & Liu, H. X. A hidden Markov model for the estimation of correlated queues in probe vehicle environments. *Transportation Research Part C: Emerging Technologies* **128,** 103128 (2021).

40. Sun, Z. & Ban, X. J. Vehicle trajectory reconstruction for signalized intersections using mobile traffic sensors. *Transportation Research Part C: Emerging Technologies* **36,** 268–283 (2013).

41. Ramezani, M. & Geroliminis, N. Queue profile estimation in congested urban networks with probe data. *Computer-Aided Civil and Infrastructure Engineering* **30,** 414–432 (2015).

42. Comert, G. & Cetin, M. Analytical evaluation of the error in queue length estimation at traffic signals from probe vehicle data. *IEEE Transactions on Intelligent Transportation Systems* **12,** 563–573 (2011).

43. Wei, H., Zheng, G., Gayah, V. & Li, Z. A survey on traffic signal control methods. *arXiv preprint arXiv:1904.08117* (2019).

44. Li, J., Yu, C., Shen, Z., Su, Z. & Ma, W. A survey on urban traffic control under mixed traffic environment with connected automated vehicles. *Transportation Research Part C: Emerging Technologies* **154,** 104258 (2023).

45. Urbanik, T. *et al. Signal timing manual* (Transportation Research Board Washington, DC, 2015).

46. Husch, D. & Albeck, J. Trafficware SYNCHRO 6 user guide. *TrafficWare, Albany, California* **11** (2004).

47. Hale, D. Traffic Network Study Tool–TRANSYT-7F, United States Version. *Mc-Trans Center in the University of Florida* (2005).

48. Chaudhary, N. & Chu, C. Passer V-Software for timing signalized arterials. *College Station, TX: Texas Transportation Institute* (2002).

49. Hunt, P., Robertson, D., Bretherton, R. & Winton, R. *SCOOT-a traffic responsive method of coordinating signals* tech. rep. (1981).

50. Lowrie, P. Scats, sydney co-ordinated adaptive traffic system: A traffic responsive method of controlling urban traffic (1990).

51. Gartner, N. H. *OPAC: A demand-responsive strategy for traffic signal control* **906** (1983).

52. Mirchandani, P. & Head, L. A real-time traffic signal control system: architecture, algorithms, and analysis. *Transportation Research Part C: Emerging Technologies* **9,** 415–432 (2001).

53. Zhang, G. & Wang, Y. Optimizing minimum and maximum green time settings for traffic actuated control at isolated intersections. *IEEE Transactions on Intelligent Transportation Systems* **12,** 164–173 (2010).

54. Wada, K., Usui, K., Takigawa, T. & Kuwahara, M. An optimization modeling of coordinated traffic signal control based on the variational theory and its stochastic extension. *Transportation research procedia* **23,** 624–644 (2017).

55. Li, W. & Ban, X. Connected vehicles based traffic signal timing optimization. *IEEE Transactions on Intelligent Transportation Systems* **20,** 4354–4366 (2018).

56. Arel, I., Liu, C., Urbanik, T. & Kohls, A. G. Reinforcement learning-based multi-agent system for network traffic signal control. *IET Intelligent Transport Systems* **4,** 128–135 (2010).

57. Khamis, M. A. & Gomaa, W. Adaptive multi-objective reinforcement learning with hybrid exploration for traffic signal control based on cooperative multi-agent framework. *Engineering Applications of Artificial Intelligence* **29,** 134–151 (2014).

58. Yau, K.-L. A., Qadir, J., Khoo, H. L., Ling, M. H. & Komisarczuk, P. A survey on reinforcement learning models and algorithms for traffic signal control. *ACM Computing Surveys (CSUR)* **50,** 1–38 (2017).

59. Chu, T., Wang, J., Codecà, L. & Li, Z. Multi-agent deep reinforcement learning for large-scale traffic signal control. *IEEE Transactions on Intelligent Transportation Systems* (2019).

60. Wu, Q. *et al.* Distributed agent-based deep reinforcement learning for large scale traffic signal control. *Knowledge-Based Systems* **241,** 108304 (2022).

61. Feng, Y., Head, K. L., Khoshmagham, S. & Zamanipour, M. A real-time adaptive signal control in a connected vehicle environment. *Transportation Research Part C: Emerging Technologies* **55,** 460–473 (2015).

62. Feng, Y., Yu, C. & Liu, H. X. Spatiotemporal intersection control in a connected and automated vehicle environment. *Transportation Research Part C: Emerging Technologies* **89,** 364–383 (2018).

63. Yu, C., Feng, Y., Liu, H. X., Ma, W. & Yang, X. Integrated optimization of traffic signals and vehicle trajectories at isolated urban intersections. *Transportation research part B: methodological* **112,** 89–112 (2018).

64. Stern, R. E. *et al.* Dissipation of stop-and-go waves via control of autonomous vehicles: Field experiments. *Transportation Research Part C: Emerging Technologies* **89,** 205–221 (2018).

65. Osorio, C. & Yamani, J. Analytical and scalable analysis of transient tandem Markovian finite capacity queueing networks. *Transportation Science* **51,** 823–840 (2017).

66. Robertson, D. I. & Bretherton, R. D. Optimizing networks of traffic signals in real time-the SCOOT method. *IEEE Transactions on vehicular technology* **40,** 11–15 (1991).

67. Derpanis, K. G. Overview of the RANSAC Algorithm. *Image Rochester NY* **4,** 2–3 (2010).

68. Wang, X. *et al.* Trajectory Data Processing and Mobility Performance Evaluation for Urban Traffic Networks. *Transportation Research Record,* 03611981221115088 (2022).

69. Saldivar-Carranza, E. *et al.* Deriving operational traffic signal performance measures from vehicle trajectory data. *Transportation Research Record* **2675,** 1250–1264 (2021).
