# Supplementary material for: Traffic light optimization with low penetration rate vehicle trajectory data
Source: Nat Commun. 2024 Feb 20;15:1306. doi: 10.1038/s41467-024-45427-4 (PMC10879128; doi:10.1038/s41467-024-45427-4)
Supplement: Supplementary file 3 — Description of additional supplementary files [file 41467_2024_45427_MOESM3_ESM.pdf]

Description of Additional Supplementary Files

Supplementary Movie 1: Demonstration of the raw vehicle trajectory data. Trajectory points are plotted over time on a map of Birmingham, MI, tracing each vehicle's movement through the network.

Supplementary Movie 2: Demonstration of Newellian coordinates & point-queue representation. This video explains how the point-queue representation using Newellian coordinates allows for lower dimensionality in traffic flow models than Eulerian and Lagrangian coordinates and is more suitable for vehicle trajectory data.

Supplementary Movie 3: Demonstration of the probabilistic time-space (PTS) diagram. The PTS diagram establishes the connection between observed vehicle trajectories and unknown traffic state parameters. In this video, an example PTS diagram is built based on certain arrival probabilities and signal states. Given the built PTS diagram, the video then shows the probability of different possible observed vehicle trajectory scenarios for a certain timestep, arrival rate, and penetration rate. Finally, the video discusses how the PTS model can be used for traffic state & parameter estimation.

Supplementary Movie 4: Demonstration of trajectory aggregation. Vehicle trajectories of the same time of day at a fixed-time signalized intersection can be aggregated into a single cycle to generate the aggregated time-space diagram. The aggregated time-space diagram can be combined to build the aggregated time-space diagram for the whole corridor. This video plots the trajectory points over time within the same cycle to show how vehicle movements along the corridor can be traced on the aggregated time-space diagram.

Supplementary Movie 5: Demonstration of the traffic state & parameter estimation. This video shows how the aggregated time-space diagram can be converted to scaled arrival and departure probability histograms, which are used to calibrate the queueing model. The penetration rate can be estimated when the model-estimated control delay is equivalent to the observed delay. The calibrated traffic flow model can be directly used for prediction given different signal timing parameters. The video also demonstrates how the method can be extended to a general network to estimate downstream arrivals given upstream departures. Given these estimates, the traffic state can be reconstructed for the entire corridor with the PTS diagram and space-mean speed heatmaps.

Supplementary Movie 6: Demonstration of pair-wise coordination diagnosis and field implementation. For each pair of intersections, the PTS model is used to see if additional changes to relative offsets can improve the coordination in terms of delay and total number of stops. The video also reports the results of the field implementation in Birmingham, MI where the average control delay and number of stops are effectively reduced.
